# Characterizing the West Nile Virus's polyprotein from nucleotide sequence to protein structure – Computational tools

**DOI:** 10.1016/j.jtumed.2024.01.001

**Published:** 2024-01-16

**Authors:** Mallari Praveen

**Affiliations:** Department of Zoology, Indira Gandhi National Tribal University, Amarkantak, Madhya Pradesh, India

**Keywords:** الأدوية المضادة للفيروسات, تصميم الأدوية, الفيروسات المصفرة, الجينوم, الفيزيوكيميائية, فيروس غرب النيل, Antiviral drugs, Drug design, Flaviviridae, Genome, Physicochemical, West Nile Virus

## Abstract

**Objectives:**

West Nile virus (WNV) belongs to the Flaviviridae family and causes West Nile fever. The mechanism of transmission involves the culex mosquito species. Infected individuals are primarily asymptomatic, and few exhibit common symptoms. Moreover, 10 % of neuronal infection caused by this virus cause death. The proteins encoded by these genes had been uncharacterized, although understanding their function and structure is important for formulating antiviral drugs.

**Methods:**

Herein, we used in silico approaches, including various bioinformatic tools and databases, to analyse the proteins from the WNV polyprotein individually. The characterization included GC content, physicochemical properties, conserved domains, soluble and transmembrane regions, signal localization, protein disorder, and secondary structure features and their respective 3D protein structures.

**Results:**

Among 11 proteins, eight had >50 % GC content, eight proteins had basic pI values, three proteins were unstable under in vitro conditions, four were thermostable according to >100 AI values and some had negative GRAVY values in physicochemical analyses. All protein-conserved domains were shared among Flaviviridae family members. Five proteins were soluble and lacked transmembrane regions. Two proteins had signals for localization in the host endoplasmic reticulum. Non-structural (NS) 2A showed low protein disorder. The secondary structural features and tertiary structure models provide a valuable biochemical resource for designing selective substrates and synthetic inhibitors.

**Conclusions:**

WNV proteins NS2A, NS2B, PM, NS3 and NS5 can be used as drug targets for the pharmacological design of lead antiviral compounds.

## الملخص

**أهداف البحث:**

ينتمي فيروس غرب النيل إلى فصيلة الفيروسات المصفرة ويسبب حمى النيل الغربي، وآلية انتقاله تكون عن طريق نوع بعوض الكيولكس. الضحايا في الغالب لا تظهر عليهم أعراض، والقليل منهم يظهر عليهم الأعراض الشائعة، 10٪ من الخلايا العصبية التي يسببها هذا الفيروس سوف تسبب الوفاة. ومع ذلك، حتى الآن لا تزال البروتينات المشفرة بواسطة هذه الجينات غير مميزة، وفي هذا الصدد، فإن فهم وظائف وبنية هذه البروتينات له أهمية في صياغة الأدوية المضادة للفيروسات.

**طريقة البحث:**

في الدراسة البحثية الحالية، تم استخدام منهجية "ان-سيليكو" مثل مجموعة متنوعة من أدوات المعلومات الحيوية وقواعد البيانات المستخدمة لتحليل بروتينات بروتينات فيروس غرب النيل بشكل فردي، والخصائص الفيزيائية والكيميائية، واكتشاف المجالات المحفوظة، وتحديد المناطق القابلة للذوبان وعبر الغشاء، وتوطين الإشارة، واضطراب البروتين وخصائص البنية الثانوية وهياكل البروتين ثلاثية الأبعاد الخاصة بكل منها.

**النتائج:**

من بين أحد عشر بروتينا، ثمانية بروتينات أعلى من خمسين بالمائة من محتوى "جي سي"، وثمانية بروتينات ذات قيم "بي إل" الأساسية، وثلاثة بروتينات غير مستقرة في الظروف المختبرية، وأربعة بروتينات ذات قيمة "ايه آي" أعلى من 100 قابلة للحرارة، وبعض البروتينات ذات قيم "جرافي" سلبية من التحليل الفيزيائي والكيميائي . تتم مشاركة جميع المجالات المحفوظة بالبروتين مع عائلة الفيروسات المصفرة. خمسة بروتينات قابلة للذوبان ولا تحتوي على مناطق غشائية. يشير بروتينان إلى توطين الشبكة الإندوبلازمية المضيفة. أظهر غير الهيكلي 2أ (إن إس) اضطرابا منخفضا في البروتين. تمثل السمات الهيكلية الثانوية وبيانات نماذج البنية الثالثة مصدرا كيميائيا حيويا قيما يوفر جزءا من المعلومات القوية لتصميم ركائز انتقائية ومثبطات اصطناعية.

**الاستنتاجات:**

يمكن استخدام بروتينات فيروس غرب النيل "إن إس 2أ" و "إن إس 2ب" و "بي إم" و "إن إس 3" و "إن إس 5" كأهداف دوائية للدراسة الدوائية لتصميم مركبات الرصاص المضادة للفيروسات.

## Introduction

West Nile virus (WNV) is an insect-borne periodic epidemic disease causing seasonal infections in temperate climatic regions. This virus belongs to the genus *Flavivirus* of the Flaviviridae family.^1^ Transmission occurs through culex species mosquitoes, which are exposed to the virus after feeding on dead birds.^2^ Transmission through breastfeeding, blood transfusion and organ transplantation have been reported to lead to infections in humans.^3^ The first infection was reported in 1937, in Uganda. In the latter half of the 20th century, the virus spread worldwide.^3^ Most infected individuals are asymptomatic, and the few symptoms include general fever, vomiting, headache and rashes. Less than 1% of affected people exhibit neuroinvasive diseases, such as encephalitis and meningitis, 10% impact on the nervous system leading to death.

The genome of WNV is composed of a positive-stranded RNA containing approximately 10,000 nucleotides.^4^ Coding regions are translated into polyproteins, which are then cleaved into structural and non-structural proteins. The structural proteins are C, PM, M and E, whereas the non-structural (NS) proteins are NS1, NS2A, NS2B, NS3, NS4A, NS4B and NS5. The C (capsid) protein packs the RNA in an immature state.^5^ The PM and M proteins are considered a single entity known as M protein, which plays a crucial role in infection by activating the viral entry proteins within the cell.^6^ The envelope formed by the E protein binds the host cell's surface receptors.^7^ E protein is typically targeted by the T (CD8+) cell response.^8^ Replication complex regulation by NS1 imparts viral viability. Host cell death, viral assembly and replication are based on NS2A protein mechanisms.^9^

The cofactor NS2B plus NS3 form the NS2B-NS3 protease complex, which plays crucial roles in polyprotein cleavage and virion replication.^5^ NS3 has a multifunctional serine protease at the NTD and a helicase at the CTD.^10^ NS3 helicase activity is regulated by NS4A, and NS3 acts as a cofactor for infectious virion propagation.^1^ NS4B blocks interferon signalling of the host cell. NS5, also known as RNA-dependent DNA polymerase, acts as a methyltransferase.^11^

WNV protease (NS2B-NS3) activity is inhibited by 8-hydroxyquinoline^12^ and the trypsin inhibitor aprotinin.^13^ Only the protease complex and E proteins have been used to design antiviral drugs by peptides and ligands. The remaining proteins have roles in replication and infection. Hence, detailed analysis of genome characteristics is a necessary prerequisite for developing drugs against WNV.

**Figure 1:**
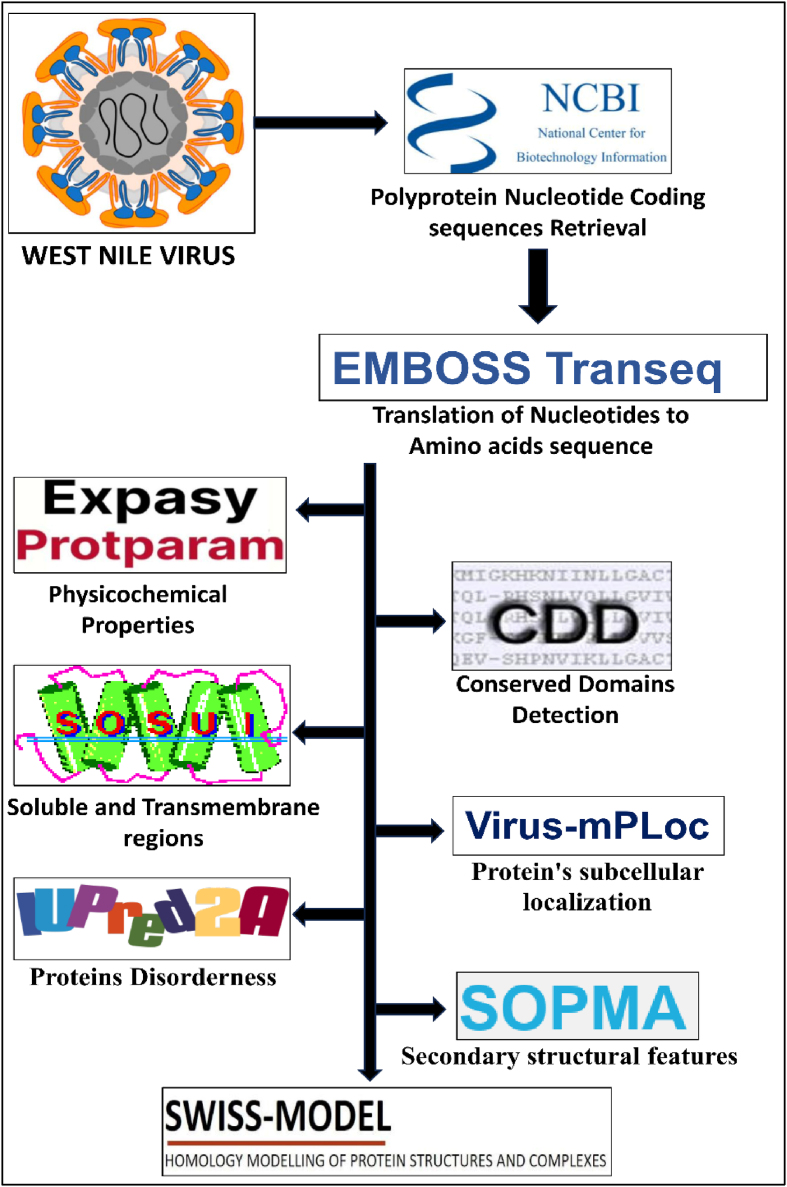
Methods used in the present study.

The present work focused on characterizing the 11,022 nucleotide WNV genome sequence retrieved from the NCBI database (https://www.ncbi.nlm.nih.gov/nuccore/KT862844.1) by using various computational tools to assess nucleotide sequence and 3D protein structure (Figure 1). To our knowledge, no prior studies have performed complete genome characterization of proteins from the WNV polyprotein. Hence, this work may help medicinal chemists design new and better antiviral drugs on the basis of the described protein properties.

## Materials and Methods

### GC content calculation and translation

The polyprotein coding nucleotide sequence of WNV was retrieved from the NCBI database (https://www.ncbi.nlm.nih.gov/) (ID-KT862844.1). The nucleotide sequence encodes 11 proteins: C, PM, M, E, NS1, NS2A, NS2B, NS3, NS4A, NS4B and NS5. The GC content of the 11 protein nucleotide coding sequences was calculated with ENDMEMO (http://www.endmemo.com/bio/gc.php), and sequences were translated with EMBOSS TransSeq (https://www.ebi.ac.uk/Tools/st/emboss_transeq/)^14^ to obtain protein sequences with standard codons and without frame shifts.

### Physicochemical properties of the proteins

The amino acid sequence of the proteins was input in ProtParam (https://web.expasy.org/protparam/) to analyse the physicochemical properties^15^; descriptors included the total number of amino acids, molecular weight, isoelectric constant (pI), total number of basic (Lys+Arg) and acidic (Glu+Asp) amino acids, extinction coefficient (EC),^16^ instability index (II),^17^ aliphatic index (AI)^18^ and grand average hydropathy (GRAVY).^19^

### Conserved domain detection

NCBI's interface was used to search conserved domain detection (CDD) data for the 11 protein sequences (https://www.ncbi.nlm.nih.gov/Structure/cdd/wrpsb.cgi).^20^ The deposited protein sequences were annotated with RPS-BLAST, a ψ-BLAST variant, to enhance the set of pre-calculated PPSSMs with query protein's, setting a threshold limit of 0.01.

### Determination of soluble or transmembrane proteins

The SOUSI server (https://harrier.nagahama-i-bio.ac.jp/sosui/mobile/) was used to determine the soluble or conserved transmembrane residues of the proteins.^21^

### Prediction of protein signal localization

The web tool Virus-mPLoc (http://www.csbio.sjtu.edu.cn/bioinf/virus-multi/), based on the data for 252 viral protein sequences, was used for predicting the viral protein's subcellular localization within the host and various sites of virus-infected cells.^22^

### Protein disorder

Under normal conditions, disordered proteins do not form well-defined tertiary structures. IUPred2A (https://iupred2a.elte.hu/),^23^ a combined web interface, was used to determine protein disorder and disordered binding regions, by using IUPred2 and ANCHOR2.

### Protein secondary structure features

Polyprotein secondary structure was determined with the web server Self Optimized Prediction Method and Alignment (SOPMA) (https://npsa-prabi.ibcp.fr/NPSA/npsa_sopma.html),^24^ which provides detailed information on the α-helix, β-sheet, random coil and extended regions of proteins, given in fasta format.

### Prediction of protein tertiary structures

Protein three-dimensional structure determination was performed through a template-based search with SWISS homology modelling (https://swissmodel.expasy.org/interactive).^25^ ProMod3 was used for structural reading of input sequences along with insertions and deletions on an alignment basis; OpenMM and OpenStructure were used for simulations; and comparative modelling followed by parameterization was performed with the CHARMM22/CMAP force field. The automated models built structures based on the updated sequences from UniProtKB and 190,687 PDB repository structures, for template identification and determination of sequence identity. Generated tertiary structures were further subjected to validation in PROCHECK with the Saves v6.0 server,^26^ to identify the percentage of residues in the allowed regions of the Ramachandran plots.

Abbreviations: 2D, Two- Dimensional/Structure; 3D, Three- Dimensional/Structure; CTD, Carboxy- Terminal Domain; EC, Extinction Coefficient; EMBOSS, European Molecular Biology Open Software Suite; ER, Endoplasmic Reticulum; GC, Guanine and Cytosine; GRAVY, Grand Average of Hydropathicity; II, Instability Index; NCBI, National Centre for Biotechnology Information; NS, Non-Structural; NTD, Nitrogen Terminal Domain; pI, Isoelectric Constant; PM, Pre-Membrane; PPSSM, Pseudo Position-Specific Scoring Matrix; RNA, Ribonucleic Acid; RPS-BLAST, Reverse Position Specific Basic Local Alignment Search Tool; WNV, West Nile Virus; QMEAN, Quantitative Model Analysis; RMSD, Root Mean Square Deviation.

## Results

### GC content calculation and translation

The total length of the WNV polyprotein was 11,022 nt; a 10,297 nucleotide coding sequence was found to encode a polyprotein (Table 1), which undergoes protease cleavage, thus forming the 11 proteins. NS5 had a nucleotide sequence length of 2715 nt, NS3 had a nucleotide sequence length of 1857 nt, M had a shorter nucleotide sequence length of 225 nt, and PM had a nucleotide sequence length of 275 nt. Among the 11 proteins, eight had >50%GC content: C, M, E, NS1, NS3, NS4A, NS4B and NS5. PM, NS2A and NS2B had 49.00–49.99 % GC content. NS4B had a high GC content of 52.15 %, and M had a GC content of 52.00 %. PM had the lowest GC content, at 49.09 %.

**Table 1:** Coding sequence length and GC content for proteins from the WNV polyprotein.

Nucleotides	Length	GC content (%)
C	369	51.49
PM	275	49.09
M	225	52.00
E	1503	51.29
NSP1	1056	50.75
NS2A	693	49.63
NS2B	392	49.23
NS3	1857	51.26
NS4A	447	51.67
NS4B	765	52.15
NS5	2715	51.82

C – Capsid; PM – Pre-Membrane; M− Membrane; E − Envelope; NS – Non-Structural Protein, GC – Guanine and Cytosine.

### Physicochemical properties of the proteins

**Figure 2:**
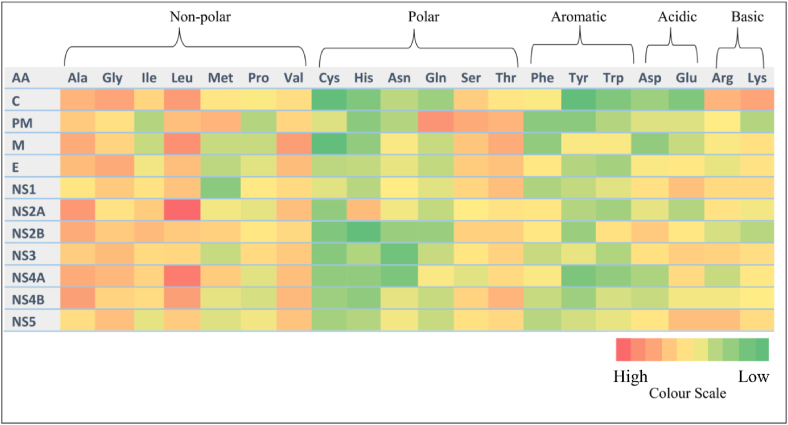
Heat map representation of amino acid composition in proteins from the WNV polyprotein.

**Figure 3:**
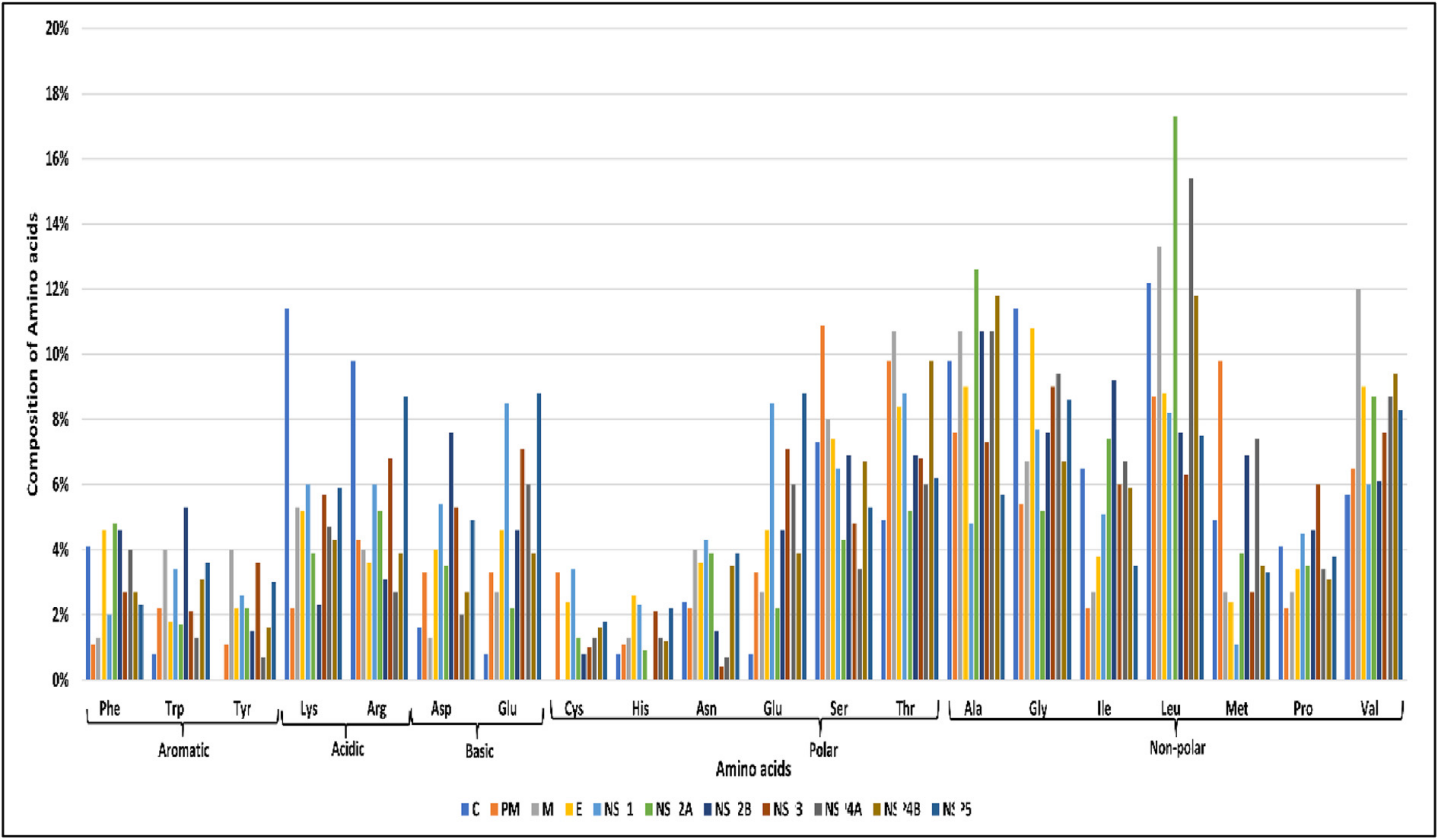
Amino acid residue composition of proteins from the WNV polyprotein.

Each protein's physicochemical properties were computed with the ExPASY ProtParam tool. Descriptors including the length of the protein, EC, molecular weight, II, pI, AI, total number of positively (Arg+Lys) and negatively (Asp+Glu) charged residues, and GRAVY values were determined (Table 2). Amino acid composition, including polar, non-polar, aromatic, acidic and basic properties, are represented in Figure 2, Figure 3.

**Table 2:** Physico-chemical properties of proteins from the WNV polyprotein.

Sl. No.	Protein	AA	M.wt.	pI	(−)R	(+)R	EC^a^	II	AI	GRAVY
1	C	123	13315.15	12.31	3	26	5500	37.66 (S)	99.19	0.030
2	PM	92	10191.73	6.72	6	6	12,615	51.77(US)	68.19	−0.185
3	M	75	8195.58	9.87	3	7	20,970	36.64(S)	107.87	0.311
4	E	501	53592.29	7.66	43	44	66,640	24.10(S)	84.07	0.053
5	NS1	352	39731.77	5.73	49	42	80,160	45.87(US)	74.20	−0.568
6	NS2A	231	25391.57	9.69	13	21	29,575	42.24(US)	133.90	0.790
7	NS2B	131	14469.98	4.11	16	7	41,480	25.97(S)	93.89	0.390
8	NS3	619	69051.48	7.32	77	77	104,655	31.28(S)	77.17	−0.449
9	NS4A	149	16062.41	6.08	12	11	12,615	33.14(S)	122.42	0.760
10	NS4B	255	27546.28	8.93	17	21	50,210	30.60(S)	107.88	0.419
11	NS5	905	103622.92	8.56	124	132	222,730	36.79(S)	72.87	−0.583

**AA** – Total Number of Amino Acids; **M.wt** – Molecular Weight; **pI** – Isoelectric Constant; (**−)R** – Total Number of Negatively Charged Residues (Asp+Glu); **(+)R** – Total Number of Positively Charged Residues (Arg+Lys); **EC** – Extinction Coefficient (^a^ units of M^−1^ cm^−1^ at 280 nm measured in water); **II** – Instability Index; **AI** – Aliphatic Index; **GRAVY** – Grand Average of Hydropathicity

The pI values for these proteins ranged from 12.31 to 4.11. Four proteins (PM, NS1, NS2B and NS4A) had acidic pI values (not less than 3), seven proteins (C, M, E, NS2A, NS3, NS4B and NS5) were basic, and C protein had a pI value of 12.31. The number of negatively and positively charged residues was more in NS5, equal in PM, and lesser in M protein. EC values at 280 nm ranged from 12,615 to 2,22,730 M^−1^ cm^−1^. PM and NS4A shared the same low EC of 12,615 M^−1^ cm^−1^. The II values ranged from 51.77 (PM) to 25.97 (NS2B). PM, NS1 and NS2A had scores >40, thus indicating their instability. The remaining eight proteins were stable, with scores <40. The AI values ranged from 107.88 (NS4B) to 68.19 (PM). M, NS2A, NS4A and NS4B had AI values > 100. The GRAVY values for PM, NS1, NS3 and NS5 were negative, ranging from −0.185 (PM) to −0.583 (NS5), whereas the remaining proteins had values of 0.030 (C) to 0.790 (NS4A).

### Conserved domain detection

In the CDD analysis of proteins from the WNV polyprotein, 4 of 11 proteins did not have domains identified from the database: PM, NS2A, NS2B and NS4B. Domains and descriptions for each protein are provided in Table 3. C, M and NS4A contained the CD of flavivirus family capsid protein, envelope glycoprotein M and the flavi_NS4 superfamily. E protein had two domain clans. Protein NS1 had a domain in the flavi_NS1 superfamily. NS3 contained three domain clans: flavivirus DEAD domain, viral helicase CTD, peptidase S7 and flavivirus serine protease NS3. NS5 contained two domain clans: one relevant to the RNA-dependent DNA polymerase, which acts as the catalytic domain in the flavivirus genus, and one specific to the Flaviviridae methyltransferase.

**Table 3:** Identified conserved domains and descriptions for proteins from the WNV polyprotein.

Protein	Domain	Description
C	Flavi_capsid	Flavivirus capsid protein C
PM	–	–
M	Flavi_M superfamily	Flavivirus envelope glycoprotein M
E	Flavi_E_C	Immunoglobulin-like domain III of Flavivirus envelope glycoprotein E
	Flavi_E_stem	Flavivirus envelope glycoprotein E, stem/anchor domain
NS1	Flavi_NS1 super family	Flavivirus NS1
NS2A	–	–
NS2B	–	–
NS3	Flavi_DEAD	Flavivirus DEAD domain
	SF2_C_viral	Viral helicase CTD
	Peptidase_S7	Flavivirus NS3 serine protease
NS4A	Flavi_NS4A superfamily	Flavivirus NS4A encoded as a single polyprotein
NS4B	–	–
NS5	Flavivirus_RdRp	RdRp catalytic core domain in Flavivirus genus.
	capping_2-OMTase Flaviviridae	Cap-0 specific (nucleoside-2′-O-)-methyltransferase of Flaviviridae

**C** – Capsid; **PM** – Pre-Membrane; **M** – Membrane; **E** – Envelope; **NS** – Non-Structural Protein.

### Determination of soluble or transmembrane proteins

**Figure 4:**
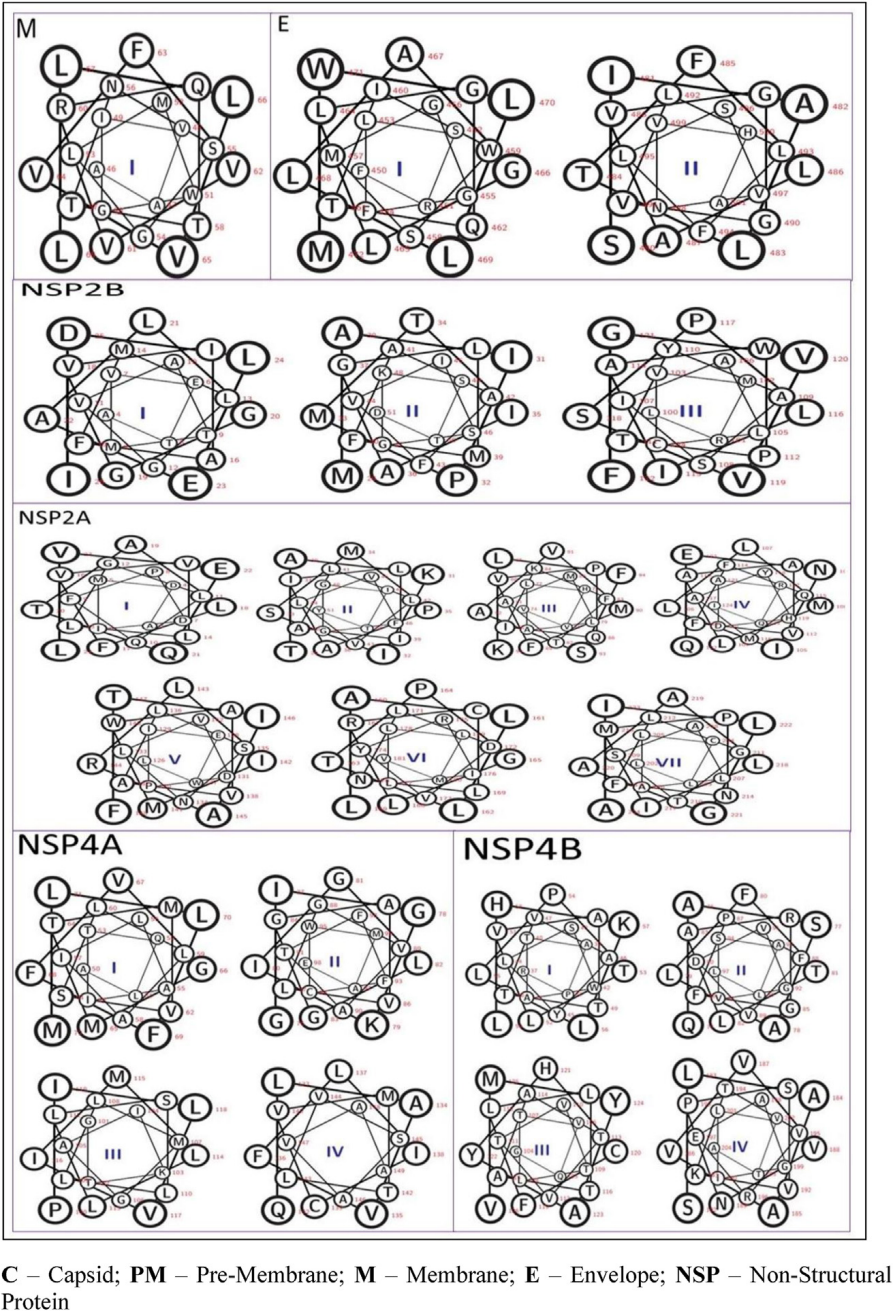
Representation of transmembrane regions in wheel form.

The SOUSI server was used to determine whether the proteins were soluble or transmembrane. M (2), NS2A (7), NS2B (3), NS4A (4) and NS4B (4) contained transmembrane regions (Table 4 and Figure 4), whereas C, PM, NS1, NS3 and NS5 did not contain transmembrane regions and were soluble.

**Table 4:** Functional characterization of proteins from the WNV polyprotein.

Protein	S/M	TM regions				Type of Peptide sequence
		NTD	Sequence	CTD	Length	
C	S	–	–	–	–	–
PM	S	–	–	–	–	–
M	M	46	AAVIGWMLGSNTTQRVVFVVLLL	68	23	Primary
E	M	450	FRSLFGGMSWITQGLLGALLLWM	472	23	Secondary
		480	SIALTFLAVGGVLLFLSVNVHA	501	22	Primary
NS1	S	–	–	–	–	–
NS2A	M	3	ADMIDPFQLGLLVVFLATQEVL	24	22	Secondary
		29	TAKISMPAILIALLVLVFGGITY	51	23	Primary
		74	VVHLALMATFKIQPVFMVASFLK	96	23	Secondary
		102	QENILLMLAAVFFQMAYHDARQI	124	23	Secondary
		126	LWEIPDVLNSLAVAWMILRAITF	148	23	Primary
		159	LALLTPGLRCLNLDVYRILLLMV	181	23	Secondary
		202	LLCLALASTGLFNPMILAAGLIA	224	23	Secondary
NS2B	M	4	ATEVMTAVGLMFAIVGGLAELDI	26	23	Primary
		29	MAIPMTIAGLMFAAFVISGKSTD	51	23	Primary
		100	LRMFCLAISAYTPWAILPSVVGF	122	23	Primary
NS3	S	–	–	–	–	–
NS4A	M	50	ALQTIALIALLSVMTMGVFFLLM	72	23	Primary
		76	GIGKIGLGGAVLGVATFFCWMAE	98	23	Primary
		101	GTKIAGMLLLSLLLMIVLIP	120	19	Primary
		132	QLAVFLICVMTLVSAVAA	149	17	Primary
NS4B	M	37	RPATAWSLYAVTTAVLTPLLKHL	59	23	Secondary
		75	QASALFTLARGFPFVDVGVSALL	97	23	Secondary
		104	GQVTLTVTVTAATLLFCHYAYMV	126	23	Primary
		182	SLAAVVVNPSVKTVREAGILVTA	204	23	Secondary
NS5	S	–	–	–		–

C – Capsid; PM – Pre-Membrane; M− Membrane; E − Envelope; NS – Non-Structural Protein; NTD – Amino Terminal Domain; CTD – Carboxy Terminal Domain; M− Membrane Protein; S – Soluble Protein; TM – Transmembrane.

### Prediction of protein signal localization

Investigation of the localization signals suggested that C, M, NS1, NS2A, NS2B, NS4A and NS4B localized in the viral capsid, whereas NS5 and NS3 localized in the viral capsid and host endoplasmic reticulum, and PM protein localized in the host cytoplasm (Table 5).

**Table 5:** Subcellular localization of the proteins from the WNV polyprotein.

Protein	Subcellular location
C	Viral capsid
PM	Host Cytoplasm
M	Viral capsid
E	Viral capsid
NS1	Viral capsid
NS2A	Viral capsid
NS2B	Viral capsid
NS3	Viral capsid, host ER
NS4A	Viral capsid
NS4B	Viral capsid
NS5	Viral capsid, host ER

### Protein disorder

**Figure 5:**
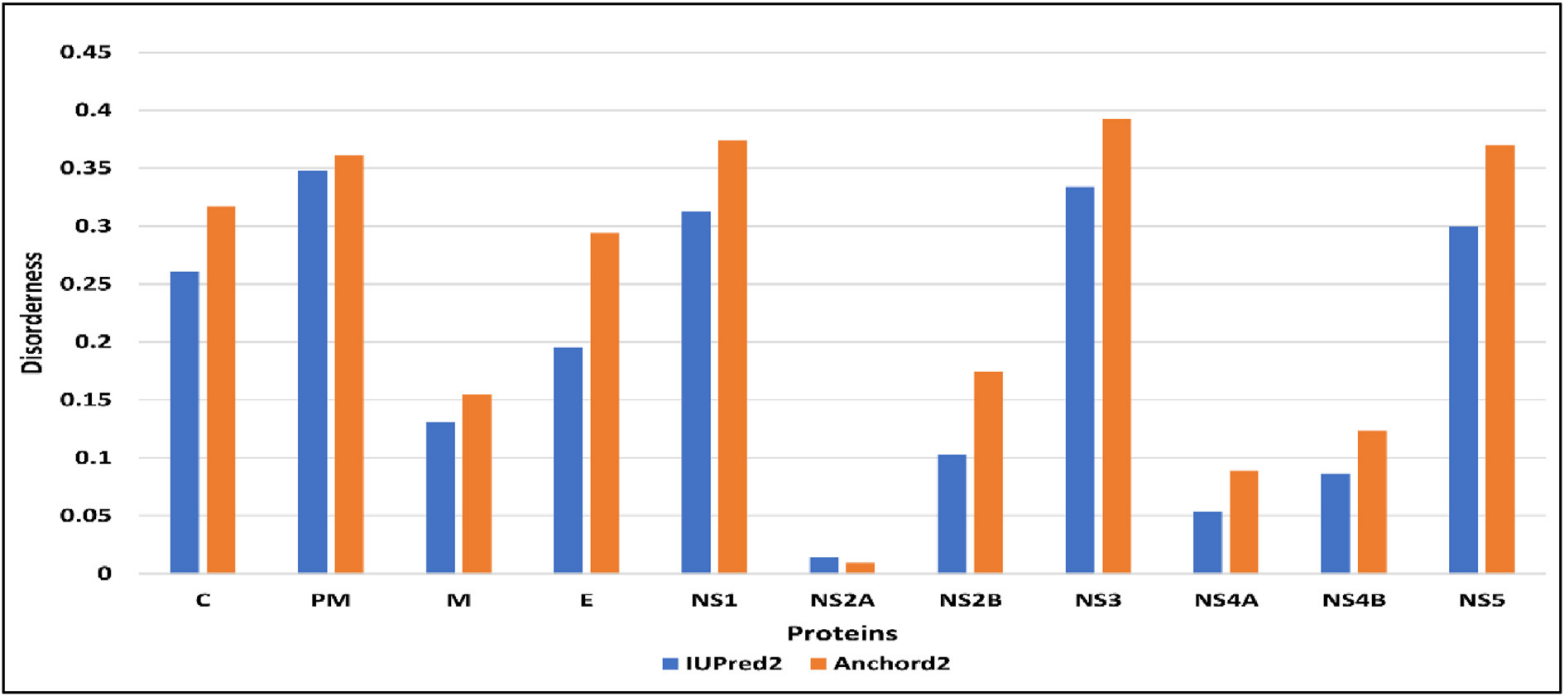
Disorder of proteins from the WNV polyprotein. IUPred2 – Disorder score of the protein region; Anchord2 – Disorder score of the binding region

Protein region disorder, as described with IUPred2 (Figure 5), indicated binding region disorder with ANCHOR2. IUPred2 analysis indicated the following in descending order: PM>NS3>NS1>NS5>C>E>M>NS2B>NS4B>NS4A>NS2A. ANCHOR2 analysis indicated the following in descending order: NS3 >NS1>NS5>PM>C>E>NS2B>M>NS4B>NS4A>NS2A.

### Protein secondary structure features

**Figure 6:**
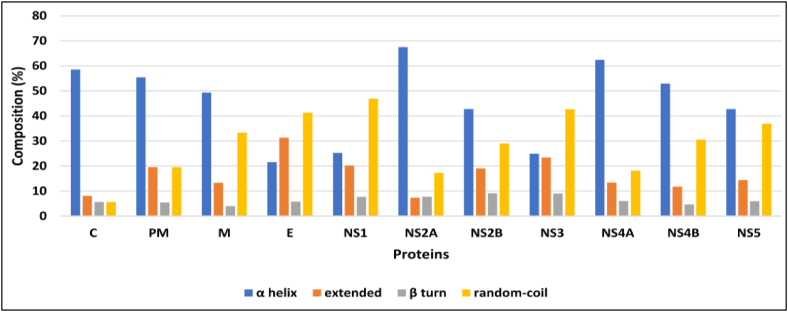
Secondary structure features of proteins from the WNV polyprotein.

Secondary features of the proteins were predicted with the SOPMA tool (composition descriptors in Figure 6). The analysis revealed that the maximum frequency of occurrence of α-helices was in NS2A (67.53), and the minimum was in E (21.56). Relatively more random coils were present in NS1 (46.88), whereas fewer were present in C (5.69). Relatively more extended turns were present in NS3 (23.42), whereas fewer were present in NS2A (7.36). The β-sheet content was relatively low in M (4.00) and high in NS2B (9.16).

### Prediction of protein tertiary structures

**Figure 7:**
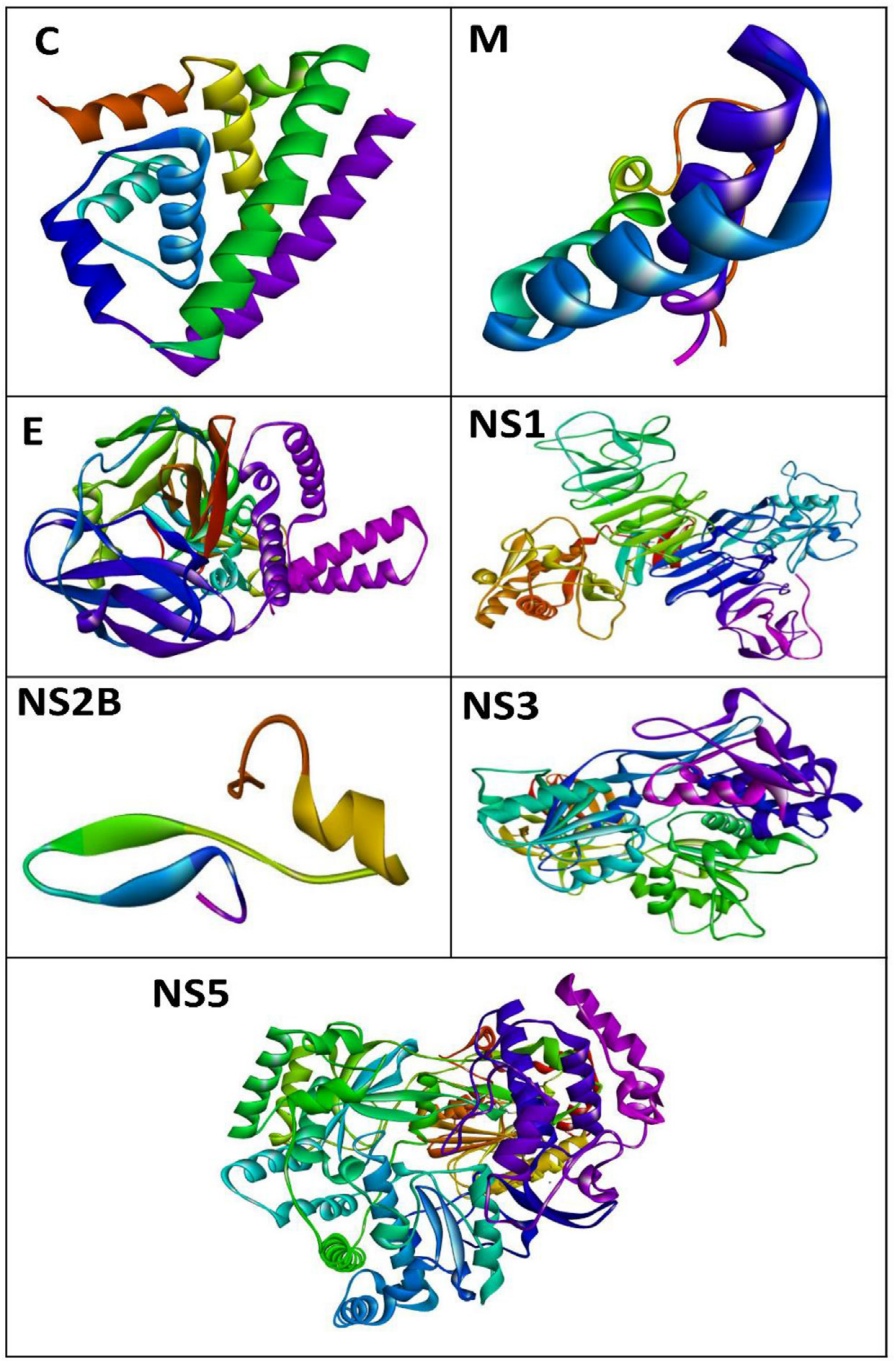
Predicted 3D structures of proteins from the WNV polyprotein.

The tertiary structures of the proteins from the WNV polyprotein models were matched to the structures deposited in the PDB (Table 6), including the percentage sequence identity and alignment sequences. The templates used were 5OW2 for C, 2BSJ for PM, 7 LCG for M, 7KV9 for E, 4TPL for NS1, 6ZLH for NS2A, 2QQV for NS2B, NS2B for NS3, 6HUM for NS4A, 5H3C for NS4B and 4K6M for NS5. A sequence identity >30 % was used to build the 3D protein structure. The built predicted 3D protein structures for C, M, E, NS1, NS2B, NS3 and NS5 are shown in Figure 7. Validation of the generated 3D protein structures with Ramachandran plots was based on >90 % of residues in allowed regions indicating the built model was appropriate. Only two proteins, NSP1 and NS2A, had <90 % of residues in allowed regions (Table 6 and Figs. S1–S11).

**Table 6:** Shared protein templates from the PDB with respect to proteins from the WNV polyprotein and validation in Ramachandran plots.

Protein	Identity (%)	PDB ID	Chain	Molecule	Alignment	Ramachandran plot allowed regions (%)
C	66.35	5OW2	AB	Japanese encephalitis capsid protein	26–98	98.4
PM	23.68	2BSJ	AB	Type 3 secretion chaperone SycT from *Yersinia enterocolitica*	7–44	92.9
M	80	7LCG	F	Mature Usutu SAAR-1776	1–75	90.9
E	97.01	7KV9	A	Chimeric flavivirus between Binjari and WNV	1–501	92.5
NS1	99.72	4TPL	AB	WNV ns1	1–352	88.4
NS2A	13.11	6ZLH	A	Glutamate receptor transporter homologue Glt Tk	51–121	86.2
NS2B	93.75	2GGV	A	WNV Serine protease subunit ns2b-ns3	50–93	94.7
NS3	79.42	2WV9	A	Ns3 protease-helicase from Murray Valley encephalitis virus	16–618	90.6
NS4A	17.72	6HUM	F	Photosynthetic complex from *Thermosynechococcus elongatus*	46–124	92.2
NS4B	16.33	5H3E	B	Isocitrate dehydrogenase 2	104–161	90.2
NS5	81.19	4K6M	A	Japanese encephalitis virus ns5	5–895	94.4

C – Capsid; PM – Pre-Membrane; M− Membrane; E − Envelope; NS – Non-Structural Protein.

## Discussion

The guanine (G) and cytosine (C) content in the nucleotide sequence affects DNA stability. A high GC content enhances genome stability at elevated temperatures and promotes strong base stacking.^27^ It imparts crucial information for determining the temperature during PCR annealing, designing primers, and constitutes a key feature in shaping proteins.^28^ The proteins NS4B, M, NS5, NS4A, C, E, NS3 and NS1 were found to have high GC content of 52.15 %, 52.00 %, 51.82 %, 51.67 %, 51.49 %, 51.29 %, 51.26 % and 50.75 %, respectively, accounting for more than 50 % of the genome; consequently, the proteins that are stable and rigid, and can withstand relatively higher temperatures than other WNV polyproteins. In PCR, when generating complementary gene sequences for these proteins, the annealing temperature is slightly higher for proteins with lower GC composition. Proteins with less stability and greater flexibility are more prone to mutations under abnormal conditions.

Physicochemical analysis was performed through an in silico approach. The pI is the pH at which no electrical charge is present on the molecule, or the total number of negative and positive charges is equal.^15^ Four proteins (PM, NS1, NS2B and NS4A) had pI values below seven, whereas the remaining seven proteins had pI values above seven. The isoelectric focusing technique is performed on the basis of the pI values to separate molecules from complexes.^29^ These values aid in the isolation of proteins of interest from the WNV polyprotein in wet laboratory experiments after digestion.

EC is defined as the amount of light absorbed per mole protein at a specific wavelength of light. A protein's EC value is calculated according to composition of tryptophan, tyrosine, and cysteine residues, because these amino acids substantially contribute to measuring the protein's optical density in the 276–282 nm range.^15^ Protein–protein and protein-ligand quantitative studies can be understood on the basis of EC values.^30^ The highest EC value was observed for the NS5 protein. II indicates protein stability under both in vivo and in vitro conditions. Proteins with II above 40 are considered unstable, whereas those with II below 40 are stable.^17^ The proteins PM, NS1 and NS2A were found to be unstable; thus, efficient procedures are required for laboratory experiments. The remaining eight proteins were found to be stable. AI is another parameter describing protein stability according to temperature. AI is defined as the relative volume occupied by aliphatic side chains, such as alanine (Ala), valine (Val), leucine (Leu) and isoleucine (Ile).^15^^,^^18^ A high AI indicates high thermostability of a protein, which is an additional factor for wet laboratory studies. Among proteins from the WNV polyprotein, NS2A (133.90) and NS4A (122.42) had high AI values indicating high stability under a wide range of temperatures. IN contrast, PM (68.15) and NS5 (72.87) had low AI values and highly flexible structures. Proteins with lower GC and AI values were more flexible, as indicated in Table 1, Table 2. GRAVY values range from –4 to +4, and indicate the hydrophilic and hydrophobic nature of proteins.^19^ A low GRAVY range suggests a globular (hydrophobic) rather than a membranous (hydrophilic) protein. In this study, the proteins with negative GRAVY values (PM, E, NS3 and NS5) (Table 2) did not form transmembrane regions, as confirmed by the SOSUI server (Table 3).

A set of proteins from the WNV polyprotein subjected to NCBI/CDD-BLAST is listed in Table 3. The proteins were found to contain conserved domains, such as Flavi_capsid, Flavi_M superfamily, Flavi_E_C, Flavi_E_Stem, Flavi_NS1 superfamily, Flavi_DEAD, SF 2_C_viral, pepridase_S7, Flavi_NS4A superfamily, Flavi_RdRp and capping_2_OMTase-Flaviviridae. Few of these domains have enzymatic activity. Four proteins, PM, NS2A, NS2B and NS4B, were found not to have conserved domains, according to the CDD search. A superfamily is a set of conserved domain models that generate overlapping annotations of protein sequences, are assumed to represent evolutionarily related domains and may be redundant with one another.^31^ The superfamily is relatively close to being identified as the Flaviviridae superfamily.

Four amino acid characteristics are applied in SOSUI predictions: hydropathy index, amphiphilicity index, amino acid sequence charge and protein length.^21^ The bilayer transmembrane functions in physiological process such as cell recognition, intracellular joining, attachment, enzymatic function and signal transduction.^32^ The results in Table 4 indicated that proteins with TM regions contained high non-polar amino acids to maintain the hydrophobicity and relatively less polar residue content. NS2A, NS4A, NS4B, NS2B, E and M contain TM regions that actively perform cellular functions.

Viruses can replicate their genome to increase their progeny only in host cells, and their functions depend on the environmental conditions in the body.^33^ Therefore, knowledge of the subcellular localization of viral proteins in host cells or virus-infected cells greatly aids in understanding the relevant mechanisms and designing antiviral drugs. On the basis of analysis of the subcellular localization of WNV proteins (Table 5), all proteins except PM were predicted to localize in the viral capsid. NS3 and NS5 were predicted to localize in the host endoplasmic reticulum, and PM protein was predicted to localize in the host cytoplasm. The translation of WNV RNA in the host ER results in polyprotein formation, increases ER stress and leads to protein unfolding.^34^

Disordered proteins have relatively low hydrophobic residue content and high amounts of charged polar residues, the latter of which are responsible for hydrophilicity and consequently interference with water molecules and the formation of weak multivariant interactions.^35^^,^^36^ Analysis of WNV polyprotein disorder (Figure 5) indicated that NS2A was less disordered and more stable than NS3, in terms of both protein regions and binding region disorder.

Among all proteins, the β-sheet composition was greatest in NS2B (Figure 6). Graces et al., in X-ray crystallography studies, have observed that NS2B comprises more β-sheets,^37^ in agreement with the results of this study. Tertiary structure has broad applications in the pharmacologic design of drugs targeting a given protein. The tertiary structures of proteins from the WNV polyprotein are represented in Figure 7. However, protein structure refinement and analysis through modelling must be performed to use these structures accurately. Nonetheless, our validation results indicated that most of the generated proteins had residues in allowed regions in the Ramachandran plot. Further structural refinements, such as QMEAN scores and RMSD, have been suggested to extend the study of these proteins. The proteins PM, NS2A, NS4A and NS4B showed <30 % identity (Table 6), and ab initio modelling must be performed to obtain their 3D structures.

In current study, we recognize the limitation of potential sampling bias, wherein the chosen proteins might not have fully captured WNV polyprotein diversity. This bias might affect the external validity or generalizability of our findings to the broader WNV population. To mitigate this limitation, future research could explore a more diverse set of proteins or consider comparisons with other WNV strains or Flaviviridae family viruses. Although this study provides valuable understanding within the scope of the selected proteins, we acknowledge the importance of expanding the analysis to gain a more comprehensive understanding.

The methods used in the current study were formulated on the basis of insights from previously published research articles.^38^^,^^39^ Although every bioinformatics tool has its own advantages, being aware of limitations is crucial. For example, working with very large datasets may lead to difficulties with EMBOSS TransSeq, thus delaying processing times. Protparam is a useful program for calculating basic protein parameters but might be unable to capture finer details, particularly in the case of sophisticated post-translational modifications or interactions with other biomolecules. Because NCBI-CDD is dependent on pre-existing databases, its accuracy relies on the comprehensiveness of those databases. Whereas SOUSI, Virus-mPLoc and IUPRED2 provide useful information about subcellular localization and protein abnormalities, they might not be able to accurately anticipate fine structural features or proteins with significant divergence. Furthermore, ANCHOR2 is useful in locating disordered binding areas, but care must be taken, because its predictions might not always align perfectly with experimental observations. SOPMA can predict secondary structures, but its accuracy varies, particularly in areas with large structural differences. SWISS-Homology modelling is a powerful tool whose efficacy is contingent on the quality and accessibility of template structures in the Protein Data Bank. Because proteins are dynamic, and computational predictions have inherent limits, results must be evaluated cautiously. These bioinformatics tools are best used in conjunction with experimental validation to support strong conclusions.

The conserved domain characteristics of WNV, in comparison to those of other flaviviruses, are highly conserved across the genus and thus may serve as potential targets for novel therapeutic strategies.^40^ A large-scale analysis of the WNV proteome has revealed the numerous evolutionarily stable nonameric positions present across the proteome and identified several completely conserved sequences.^41^ Mutational studies on the fusion glycoproteins indicated they inhibited the Zika virus and yellow fever virus, but not WNV, in terms of production of infectious virions. These conserved sequences are shared by other flaviviruses and have been associated with the functional and structural properties of viral proteins.^42^ Additionally, the WNV envelope glycoprotein fusion peptide region has been identified as an immunodominant epitope stimulating antibodies, particularly monoclonal antibodies with diverse patterns of cross-reactivity.^43^ Understanding these conserved domain characteristics is important for the development of antiviral therapies and the design of peptide-specific vaccines for flavivirus infections.^44^

Because in silico methods were used, additional study limitations include the valid lack of wet laboratory (in vivo and in vitro) investigation. The computational tools and servers, after updating or reconstruction with various algorithms, might yield different results even if the same sequence of the WNV is input. Future investigations based on the key findings of this study might consider the non-structural and pre-membrane proteins of the WNV polyprotein. Notably, NS2A, NS2B, PM, NS3 and NS5 proteins may be favourable drug targets for designing small molecules/ligands in drug discovery^45^ and epitope design through immunoinformatic approaches.^46^

## Conclusion

The proteins derived from the WNV polyprotein are associated with viral replication and induce disease in host cells. In this present study, several bioinformatic tools were used to study the characteristics of the WNV genome, including basic nucleotide sequences and complex 3D protein structures for structural and non-structural proteins. NS4B protein, with its high GC content, is considered relatively stable. In physicochemical analysis, the pI of C protein was highly basic, whereas that of NS2B was weakly acidic. The total number of positive and negative amino acid residues was relatively greater in NS5, thus indicating its high reactivity and ability to be isolated easily from the complex, given its high EC value. NS2A was the most thermostable among all proteins, on the basis of its high AI. Four proteins did not have transmembrane domains, on the basis of negative GRAVY values. Conserved domains were identified from the Flaviviridae family. The SOSUI server identified seven proteins with transmembrane domains. The 2D and 3D structures of these proteins provide insights for developing accurate models. Therefore, these proteins might be crucial for host invasion and therefore could potentially be used as drug targets for pharmacological study.

## Source of funding

This research did not receive any specific grant from funding agencies in the public, commercial or not-for-profit sectors.

## Conflict of interest

The author declares that there are no conflicts of interest regarding the publication of this article.

## Ethical approval

There are no ethical issues.

## Authors contributions

The author has critically reviewed and approved the final draft, and is responsible for the content and similarity index of the manuscript.

## Declaration of generative AI and AI-assisted technologies in the writing process

During the preparation of this work, the author used ChatGPT and QuillBOT to improve the language. After using this tool/service, the author reviewed and edited the content as needed and takes full responsibility for the content of the publication.
