# Supplementary material for: Characterizing the West Nile Virus's polyprotein from nucleotide sequence to protein structure – Computational tools
Source: J Taibah Univ Med Sci. 2024 Jan 16;19(2):338–50. doi: 10.1016/j.jtumed.2024.01.001 (PMC10831166; doi:10.1016/j.jtumed.2024.01.001)

Figure S1. Ramachandran Plot of the C protein


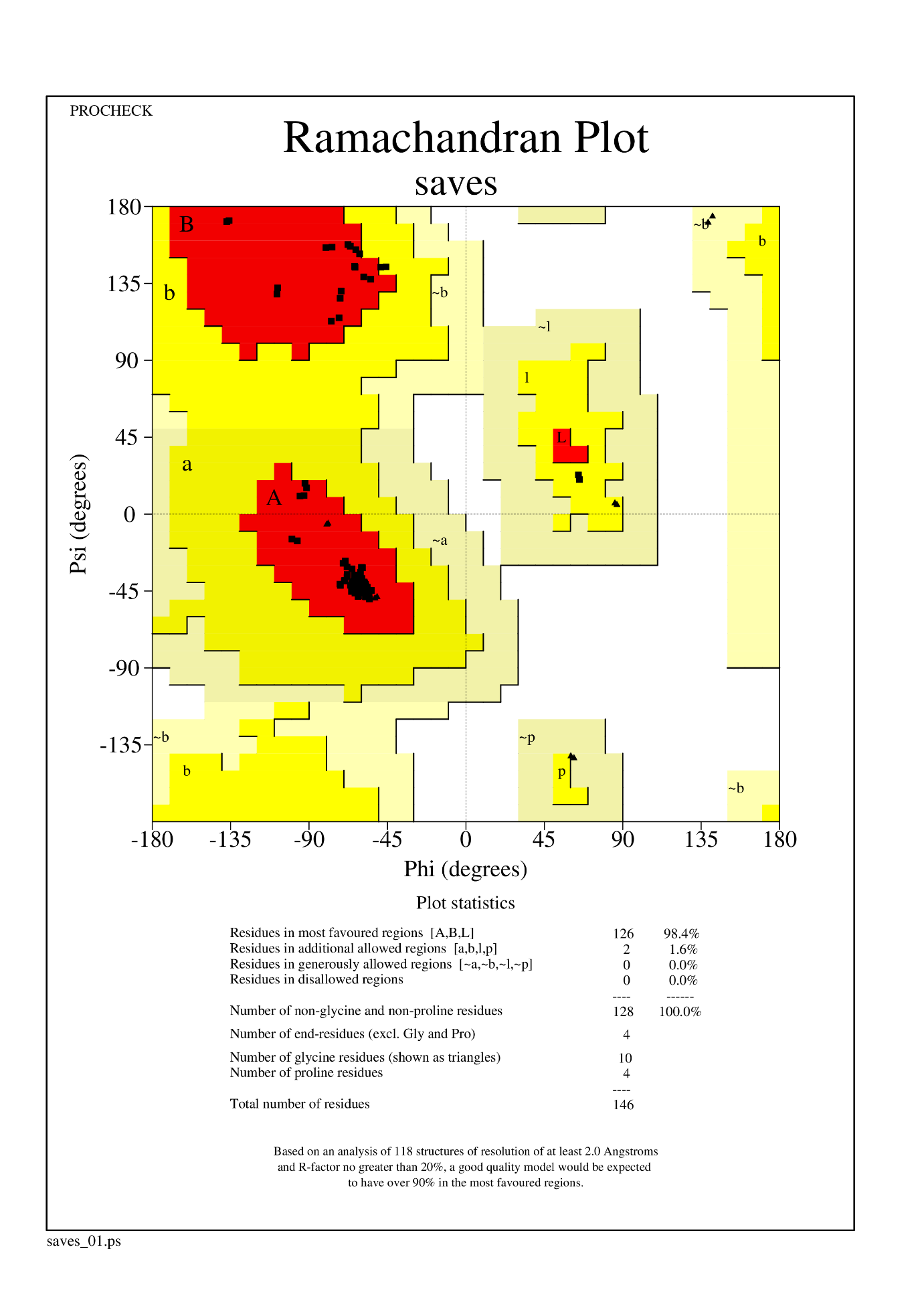


Figure S2. Ramachandran Plot of the E protein


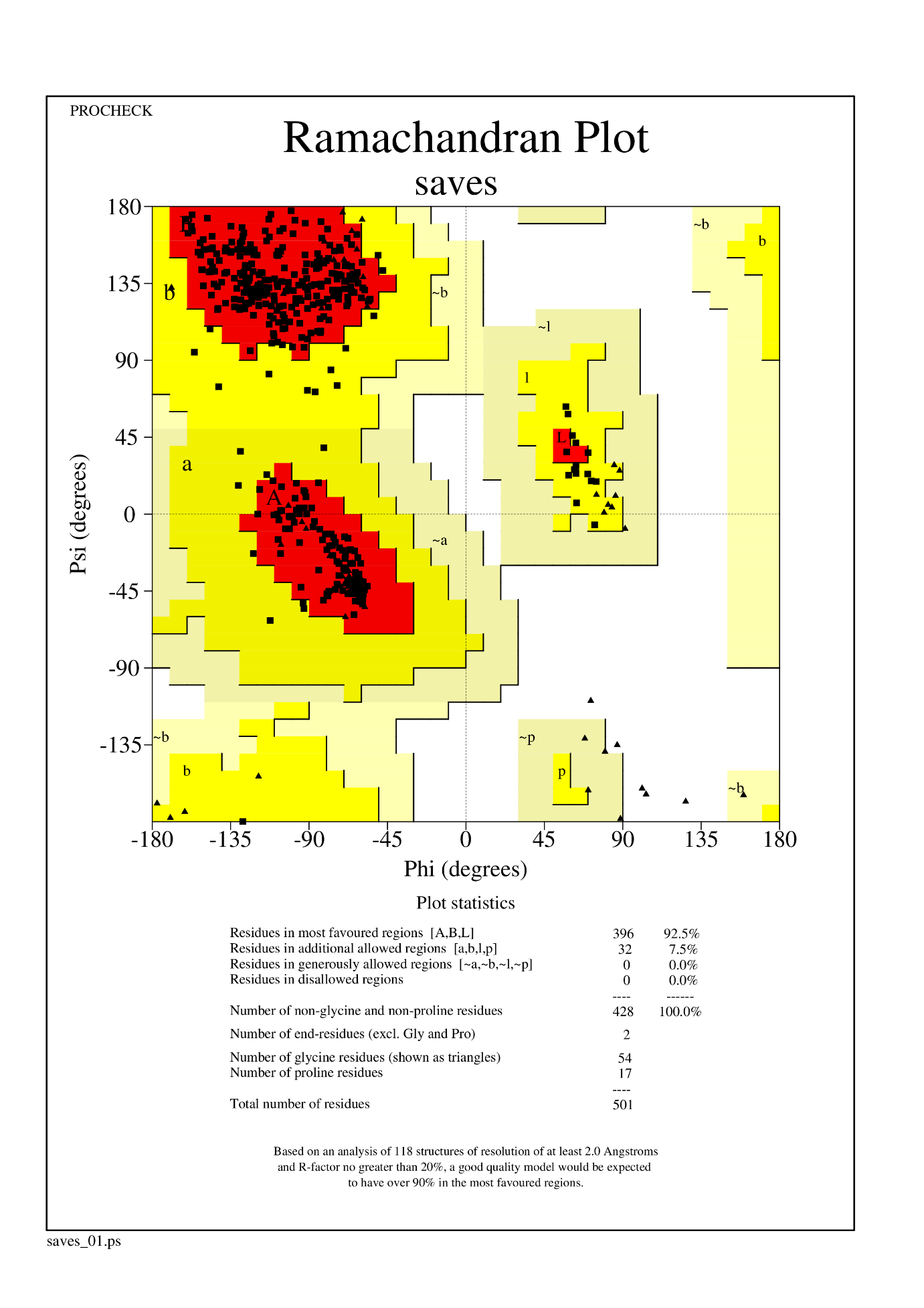


Figure S3. Ramachandran Plot of the M protein


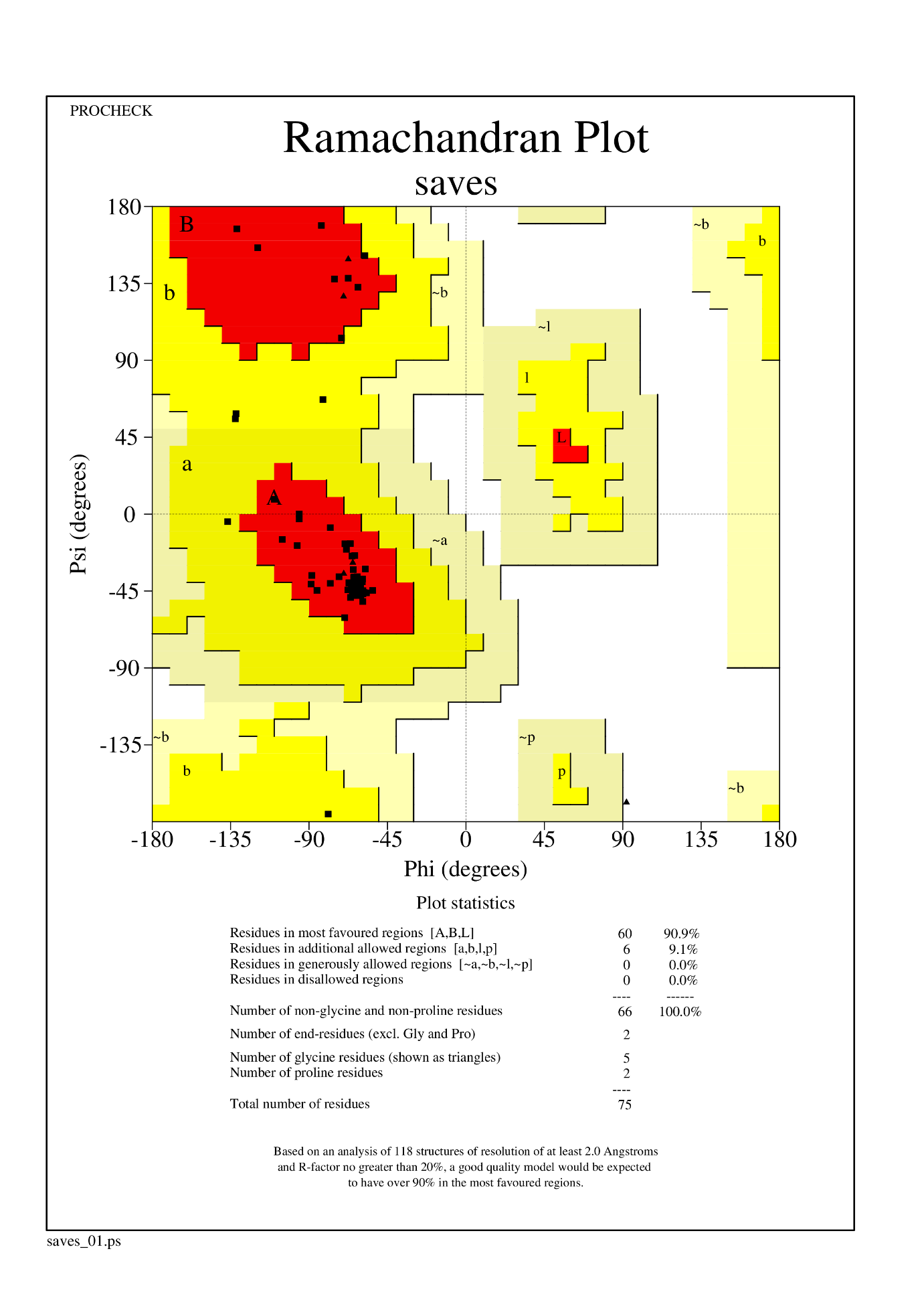


Figure S4. Ramachandran Plot of the NS1 protein


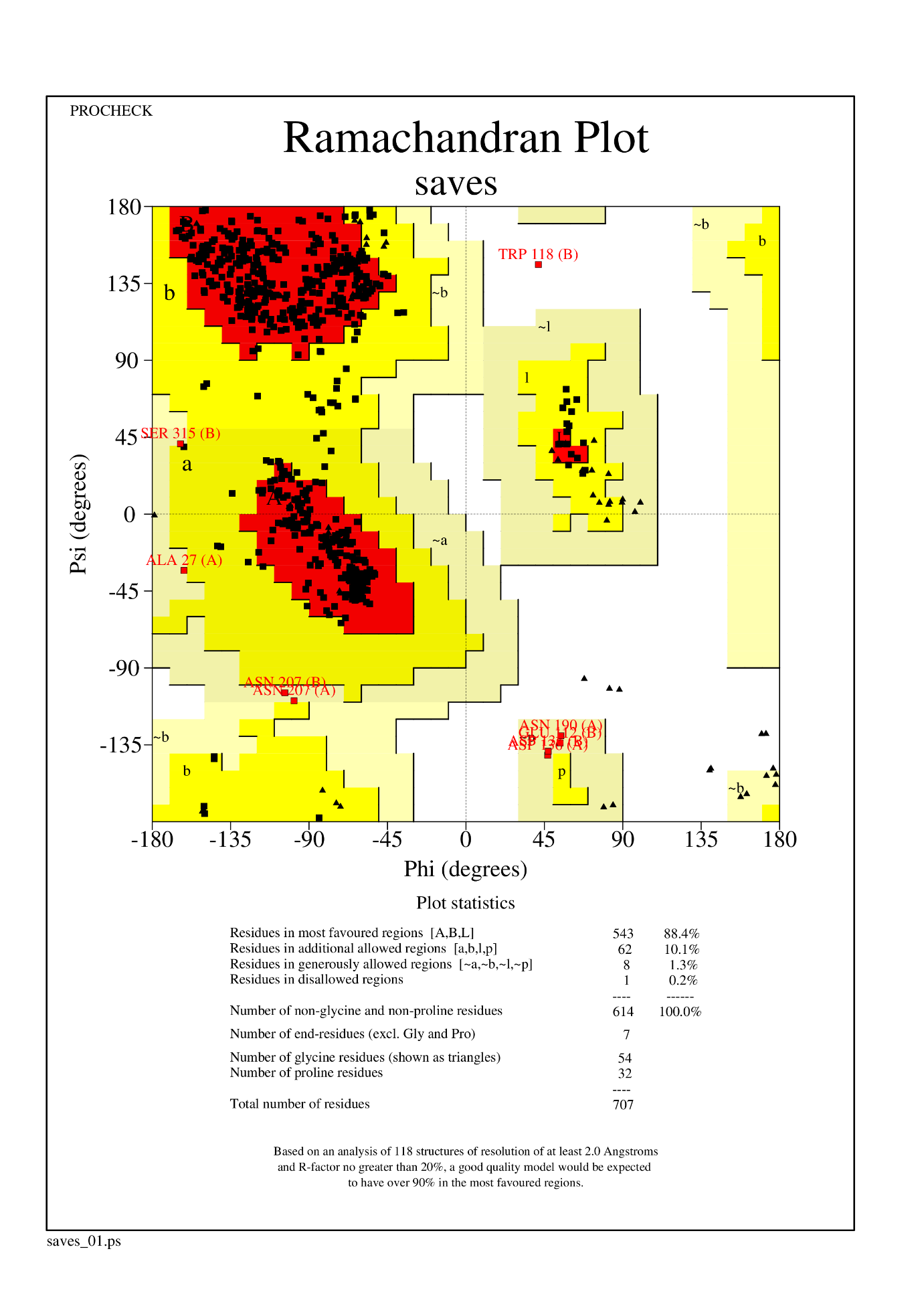


Figure S5. Ramachandran Plot of the NS2A protein


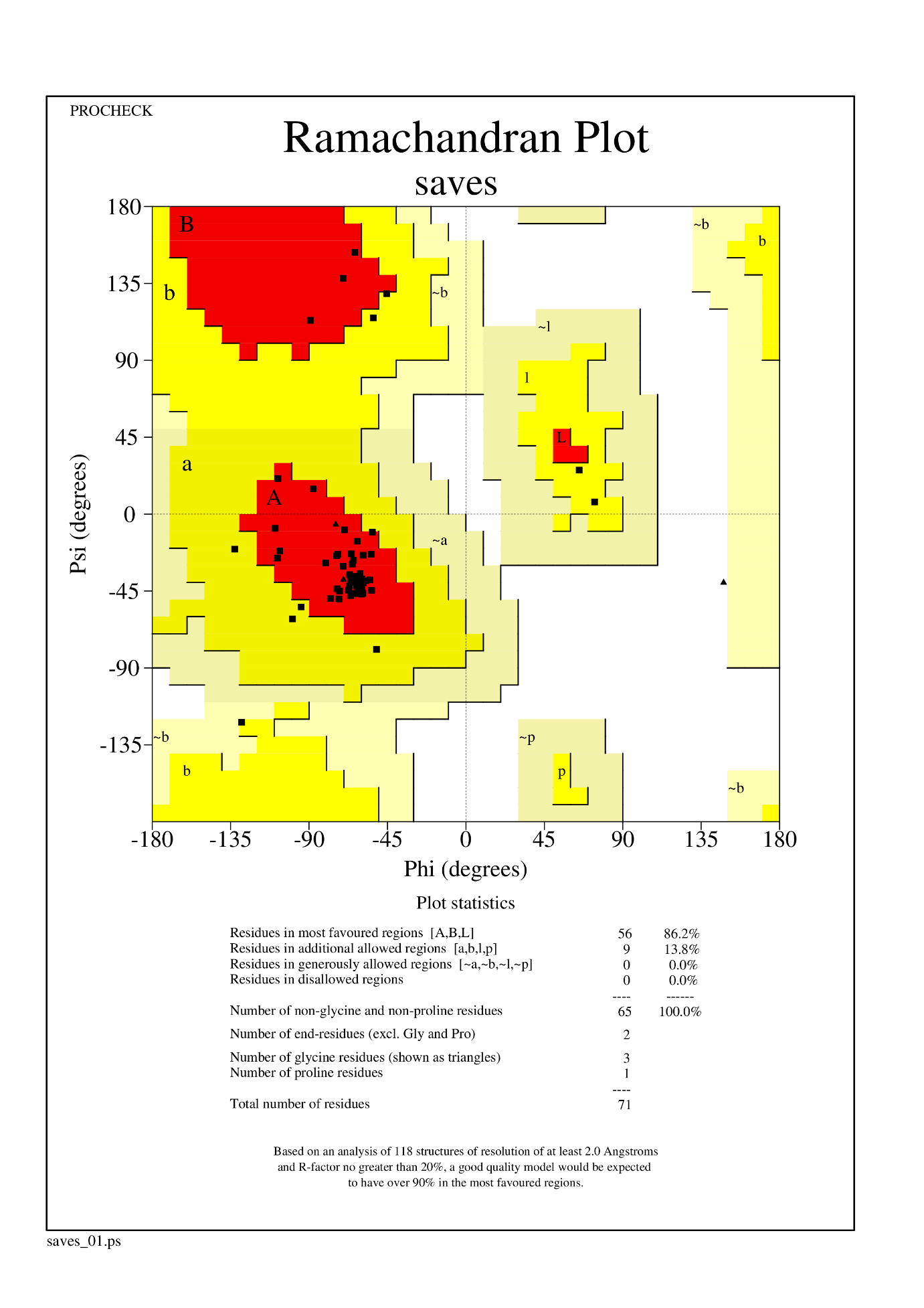


Figure S6. Ramachandran Plot of the NS2B protein


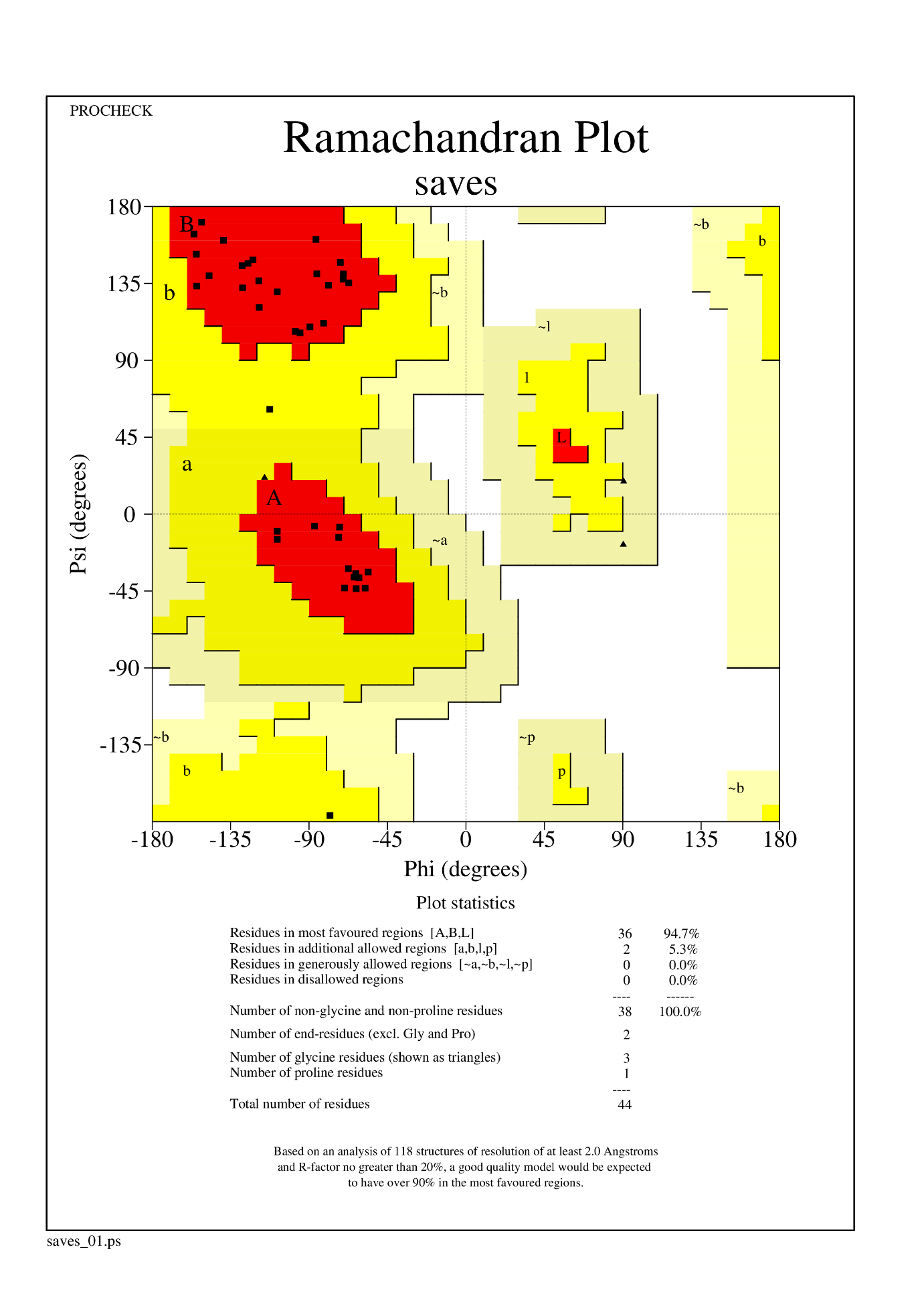


Figure S7. Ramachandran Plot of the NS3 protein


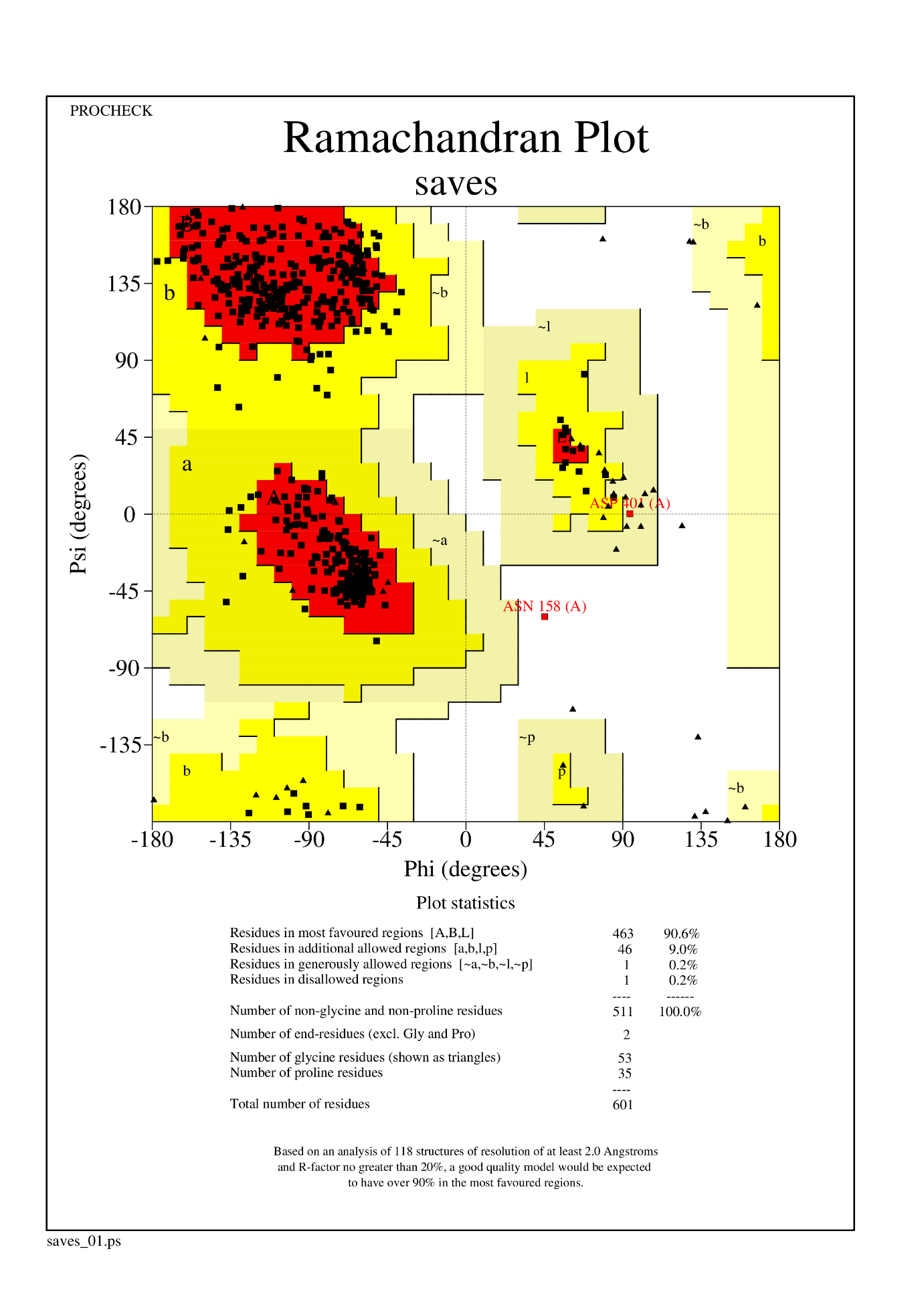


Figure S8. Ramachandran Plot of the NS4A protein


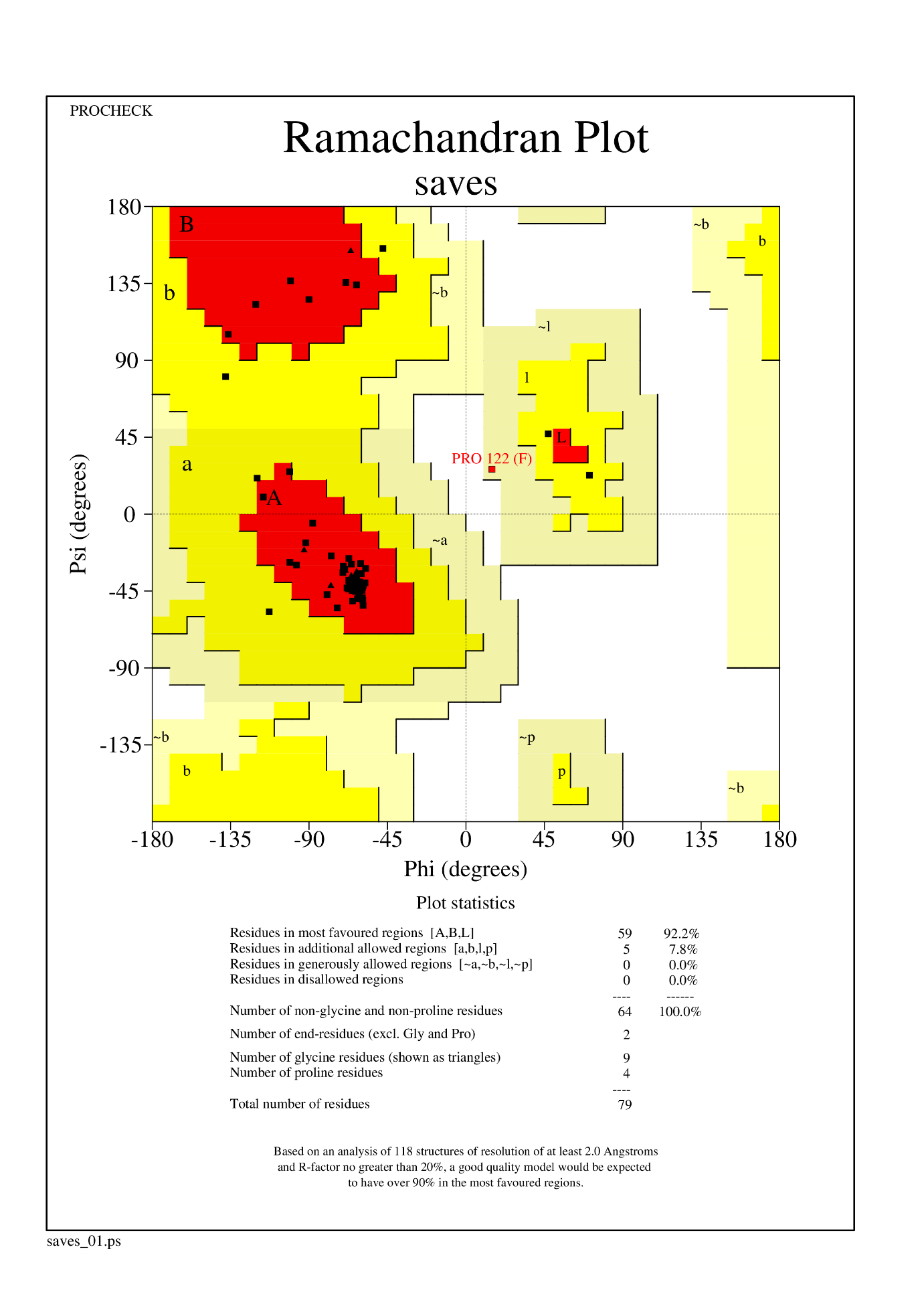


Figure S9. Ramachandran Plot of the NS4B protein


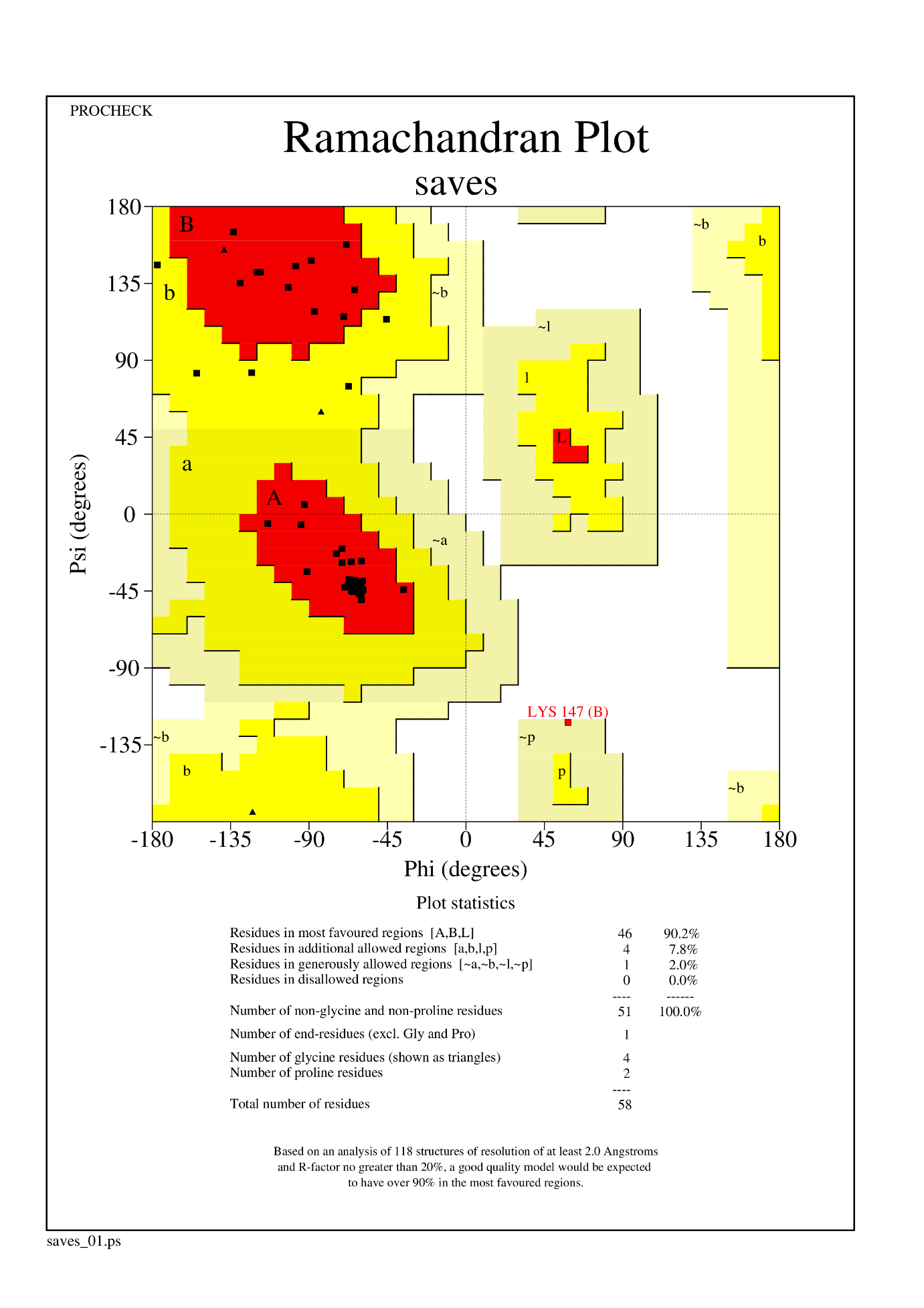


Figure S10. Ramachandran Plot of the NS5 protein


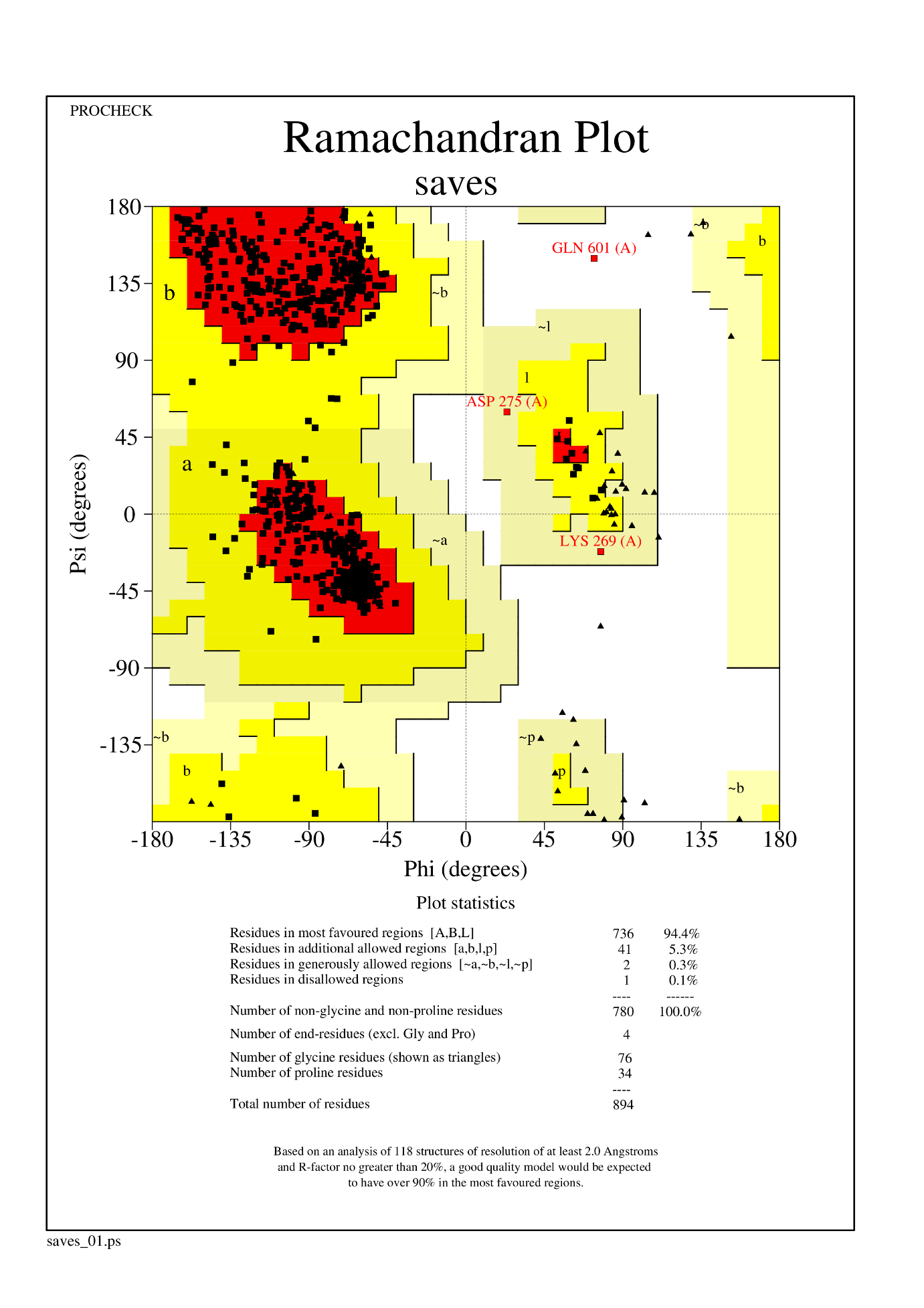


Figure S11. Ramachandran Plot of the PM protein


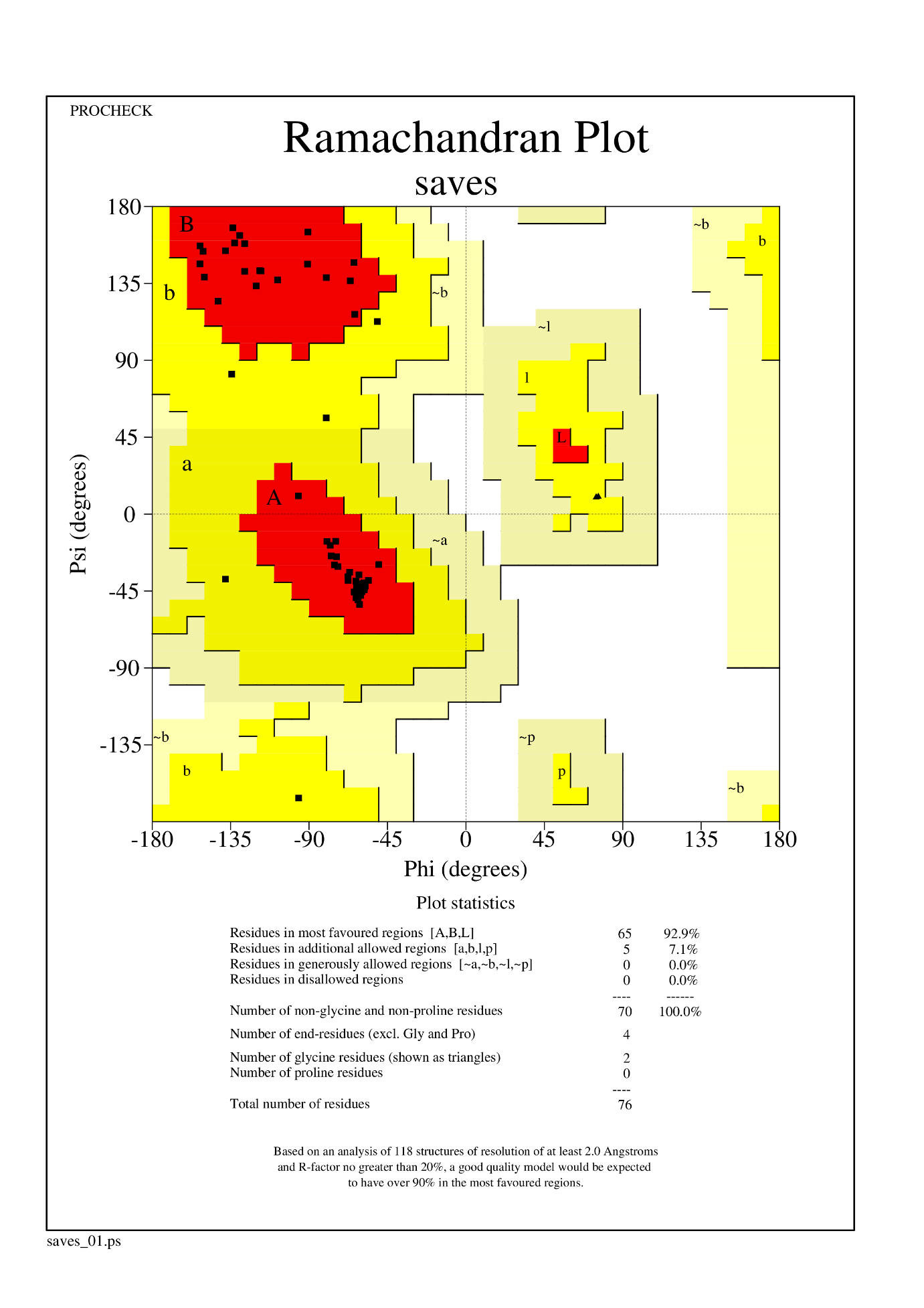

Supplement: Multimedia component 1 [file mmc1.docx]
